# Optical Characterization and Prediction with Neural Network Modeling of Various Stoichiometries of Perovskite Materials Using a Hyperregression Method

**DOI:** 10.3390/nano12060932

**Published:** 2022-03-11

**Authors:** Soo Min Kim, Syed Dildar Haider Naqvi, Min Gu Kang, Hee-eun Song, SeJin Ahn

**Affiliations:** 1 Nano Electronic Materials & Components Research Center, IT Materials & Components Research Division, Gumi Electronics & Information Technology Research Institute (GERI), Gumi 39171, Korea; smkim83@geri.re.kr; 2 Photovoltaics Research Department, Korea Institute of Energy Research (KIER), Daejeon 34129, Korea; dildar@kier.re.kr (S.D.H.N.); mgkang@kier.re.kr (M.G.K.); 3 Department of Renewable Energy Engineering, University of Science and Technology (UST), Daejeon 34113, Korea

**Keywords:** perovskite, stoichiometry, optical properties, ellipsometry, neural network, deep learning, hyper regression, backpropagation

## Abstract

Quaternary perovskite solar cells are being extensively studied, with the goal of increasing solar cell efficiency and securing stability by changing the ratios of methylammonium, formamidinium, I_3_, and Br_3_. However, when the stoichiometric ratio is changed, the photoelectric properties reflect those of different materials, making it difficult to study the physical properties of the quaternary perovskite. In this study, the optical properties of perovskite materials with various stoichiometric ratios were measured using ellipsometry, and the results were analyzed using an optical simulation model. Because it is difficult to analyze the spectral pattern according to composition using the existing method of statistical regression analysis, an artificial neural network (ANN) structure was constructed to enable the hyperregression analysis of n-dimensional variables. Finally, by inputting the stoichiometric ratios used in the fabrication and the wavelength range to the trained artificial intelligence model, it was confirmed that the optical properties were similar to those measured with an ellipsometer. The refractive index and extinction coefficient extracted through the ellipsometry analysis show a tendency consistent with the color change of the specimen, and have a similar shape to that reported in the literature. When the optical properties of the unmodified perovskite are predicted using the verified artificial intelligence model, a very complex change in pattern is observed, which is impossible to analyze with a general regression method. It can be seen that this change in optical properties is well maintained, even during rapid variations in the pattern according to the change in composition. In conclusion, hyperregression analysis with n-dimensional variables can be performed for the spectral patterns of thin-film materials using a simple big data construction method.

## 1. Introduction

Lead–halogen perovskite materials are being increasingly used in solar cells as light absorbers. Such materials were first reported in 1978, and their utility in opto-electronic fields has been studied by applying them to devices such as photodetectors and lasers [1,2,3,4,5,6]. Owing to the advantageous optical and electrical properties of these materials, they have been the focus of considerable interest, especially in the photovoltaic industry. The first solar cell manufactured using the organic–inorganic lead halide perovskite compounds CH${}_{3}$NH${}_{3}$PbBr${}_{3}$ and CH${}_{3}$NH${}_{3}$PbI${}_{3}$ was reported to have an efficiency of 3.81% [7]. Although the efficiency of the first perovskite solar cell was very low, the photovoltaic effect was confirmed. Various syntheses and device structures have been investigated to increase the power-generation efficiency of perovskite solar cells. The highest efficiency recorded in this structure was 25.7%, in which reliability was ensured even after 500 h [8,9,10,11,12,13,14]. Inexpensive solar cells can be manufactured if perovskite solar cell efficiency can be increased and its reliability improves. Hence, various studies related to perovskite have been conducted. Recently, multi-junction solar cells have been studied to maximize their power conversion efficiency using perovskite materials [15].

Currently, the most widely used perovskite material for manufacturing solar cells is methylammonium lead iodide (CH${}_{3}$NH${}_{3}$PbI${}_{3}$). This is a metal halide perovskite with a cubic crystal structure, and is expressed as ABX${}_{3}$. A solar cell composed of such a perovskite material has limited power conversion efficiency. When exposed to moisture, MAPbI${}_{3}$ material forms monohydrate and dihydrate, and finally decomposes into PbI${}_{2}$. It has been reported that it is easily decomposed even at 85 ${}^{\circ }$C or higher [16,17,18]. To solve this problem, research using other organic ions was attempted by replacing methyl ammonium (MA; CH${}_{3}$NH${}_{3}$) with suitable materials. An alternative organic ion typically used in perovskite ion replacement studies is formamidinium (FA; CH(NH${}_{2}$)${}^{2+}$). FAPbI${}_{3}$ material crystallizes above 150 ${}^{\circ }$C and changes from a hexagonal structure (yellow δ–phase) to a trigonal structure (black α–phase) [19]. The use of FA organic ions improves the power conversion efficiency of solar cells [20,21]. However, it has been reported that the color of the FA material changes with time, resulting in the deterioration of solar cell characteristics, which lowers the stability of perovskite solar cells [22]. To solve this problem, a study was conducted to increase efficiency and stability using a mixture of MA and FA materials [23]. Similarly, recent studies have replaced iodine with other substances, mainly bromide [24]. Currently, perovskites with satisfactory optoelectronic properties are produced with the generic chemical formula of MA${}_{x}$FA${}_{1-x}$Pb(I${}_{y}$Br${}_{1-y}$)${}_{3}$, defined by the quaternary stoichiometric ratio.

Generally, the perovskite material is deposited using a spin-coating method on a glass substrate coated with ITO after the stoichiometric ratio is adjusted using a liquid precursor. Thereafter, the deposited perovskite is transformed and grown into a multi-crystalline phase during the solidification process. However, this irregularly changes the chemical composition inside the perovskite over the entire deposited layer. This non-uniformity of the chemical composition inside the perovskite material creates differences in the chemical properties of each crystal in the crystallized perovskite. These results show that, unlike general single-crystal materials, crystallized perovskite materials have statistical chemical composition, making it difficult to accurately define their chemical properties. The statistical chemical composition characteristics of quaternary perovskite create variations in the experimental conditions depending on the researcher, manufacturing environment, and process sequence. Owing to these variable conditions, it is very difficult to accurately achieve the target chemical composition of the perovskite.

Typically, to determine the optimal conditions for a thin-film solar cell, it is necessary to accurately check the optoelectronic properties of the materials constituting the individual layers. When the information regarding these photoelectric properties is inaccurate or insufficient, it is necessary to check the power conversion efficiency by manufacturing thin-film solar cells for all chemical compositions of the perovskite. To reduce the amount of experimentation, numerical simulations must be performed with accurate information about the materials applied to the manufacturing equipment to predict the potential performance of certain process variables. From an optical point of view, the optimal thickness of the perovskite material for absorbing sunlight must be considered to increase the performance of thin-film solar cells in numerical simulations. In addition, the optical diffraction characteristics due to the difference in refractive index between the hole transport layer, electron transport layer, and ITO thin films in contact with the perovskite should be considered. In particular, the electrical conductivity of perovskite materials depends on the direction of growth of the crystals. Therefore, from the electrical point of view of the thin-film solar cell, applying a thick perovskite may increase the overall resistance and deteriorate the performance of the device. In numerical simulations, the necessary details to determine the optimal thickness of the light absorption layer are the refractive index and extinction coefficient according to the change in the wavelength of the perovskite material.

Considering these factors, it can be said that the fabrication of a stable and reliable perovskite is essential for research. However, the physical properties of quaternary perovskite materials vary according to the environmental conditions in the research [22]. In particular, because the environmental variables applied in the perovskite solidification process are very diverse, it is difficult to manufacture the same perovskite material as reported in literature if the environmental conditions and apparatus differ from those in the original research. Hence, it is generally difficult for researchers to use the reported composition values interchangeably, because there may be differences in the chemical composition of the final perovskite product, even if the same experimental process as that reported in the literature is followed. In summary, because the optimal stoichiometric ratio in the process of manufacturing a quaternary perovskite solar cell depends on the research environment, it is recommended that researchers not apply results reported in literature without verification; instead, they must fabricate a device for each chemical composition under the same environmental conditions and evaluate its performance. However, in reality, it is very difficult for an individual researcher to conduct studies under all conditions; hence, a method for predicting the physical properties of the quaternary perovskite depending on changes in composition is required. If the distribution of optical properties of perovskite is defined through reliability-based prediction of physical properties, a different chemical composition can be selected according to the purpose of light absorption of each solar cell. For example, even in the case of a solar cell using a single junction and multiple junctions, the composition of the perovskite optimized for each condition is different because the light absorption target changes. To select the chemical composition of perovskite to optimize the optical properties of each solar cell, experimental data for all compositions are required. However, it is very difficult to fabricate and analyze a solar cell because the conditions of these experiments are very diverse. The prediction of these properties enables the efficient design of experiments, and reliable optimal points can be identified in the environments of individual researchers.

The method of identifying a trend based on discrete data is widely used in the fields of natural science and data science. If this tendency is identified and expressed in the form of a linear equation, it is possible to predict the change before and after the current point in time. However, it is difficult to establish a regularity that can satisfy a linear equation for the optical pattern characteristics of each spectrum, expressed using the refractive index and extinction coefficient. From the human cognition point of view, this irregular change in the spectrum can be recognized as having a certain tendency; however, expressing the irregularity as a linear equation requires high-level mathematical skills and exceptional conditions. The creation of such a mathematical model requires considerable research effort. Moreover, because the constructed model can predict only in a very limited range, a wide range of models suitable for the various environments of individual researchers needs to be generated, which is difficult. Therefore, a technology based on machine learning, which is currently widely used in data science, is required to define the regularity based on the changes in the irregular physical properties of perovskite and predict its properties according to the changes in the chemical composition.

Currently, machine learning-related research is being actively conducted, and many results have been reported in the fields of traffic speed prediction, medical image screening, and wind speed forecasting [25,26,27]. In the field of materials science, it has been shown that the material design process can be drastically reduced using machine learning algorithms [28,29,30,31,32]. A study was conducted by the photovoltaic industry to predict the environmental conditions and performance of a power plant in real time using machine learning technology [33,34]. Machine learning-based methods have been applied to the photovoltaic industry to develop a forecasting technology for a target result to be achieved in a specific period [28,35,36]. Nasim et al. reported the results of applying neural network learning by constructing big data as a result of simulating the power conversion efficiency according to the change in the thin film thickness and energy bandgap of the perovskite solar cell in the SCAPS-1D program [35]. It is impossible to predict multidimensional data such as optical properties, because only simple values can be predicted in the form of conversion efficiency, which is scalar data, for a specific input thin film condition. In the case of Nelson et al., the optical characteristics according to the arrangement of perovskite nano particles were learned by artificial intelligence using the results of ï﹁nite difference time domain (FDTD) simulation, and the results showed low reliability [36]. Since these methods basically use equation-based simulation programs, big data configuration is possible, but problems occur in the definition of the neural network learning model and data, and thus show low reliability. Convolutional neural network (CNN) technology is widely applied in the development of forecasting technology in the solar industry [28]. It is mainly used for two-dimensional images, and is capable of detecting internal objects and classifying them according to their features Although this method is suitable for image processing, it is too complicated to be used for predicting optical properties in the field of materials science. To solve this problem, we need to rebuild the fundamental principles of machine learning technology. In this study, a big data structure for learning a predictive model is first defined using optical spectral data extracted from actual fabricated perovskite. This is the world’s first implementation of hyperregression technology that can easily build high-reliability artificial intelligence models from big data.

Considering the principle of the basic error backpropagation algorithm in the machine learning method, the neural network route with the least error is selected to discover the association between the observable variables. This process is similar to the linear regression commonly used in statistics. Regression analysis is used to explain the linear relationship between two-dimensional discrete data, and as the shape of the data becomes increasingly complex, the analysis is performed using a polynomial regression model. This analysis is verified through a visualization process using graphs, called curve fitting. Because the statistical regression method defines the relationship between the variables, it is difficult to visualize the three-dimensional or higher-order data, which makes it difficult to apply the regression equation. To solve this problem, it is necessary to develop a technique for selecting the neural network route with the least error for high-dimensional data using machine learning technology.

Because numerous types of variables are related to the optical properties of perovskite materials, the properties can be predicted using machine learning technology by processing the optical property information with n-dimensional variables. In this study, we developed a regression technique using an ANN, which can analyze the relationship between variables in a high-dimensional data structure such as in hyperregression analysis. The characteristic of hyperregression analysis is that only the prediction within the measured boundary range is valid; therefore, it is useful for data analysis within a certain composition range where the physical limits are defined, such as the stoichiometric ratio. In this study, a quaternary perovskite material was prepared, and the optical properties were analyzed according to the stoichiometric ratio. Through ellipsometry analysis, the refractive index and extinction coefficient of the perovskite material were extracted according to the stoichiometric ratio and visualized according to the change in the quaternary system. In addition, the optical properties of the perovskite materials were predicted through hyperregression analysis in a three-dimensional or higher-order data structure using the error backpropagation algorithm in an ANN structure. The hyperregression analysis developed in this study can be used to predict the characteristics even in an environment in which various n-dimensional variables are applied. This will enable researchers to develop artificial intelligence for predicting physical properties, because an adequate amount of big data can be collected even with a small amount of data that would be deemed insufficient for learning in optical spectrum pattern analysis using the CNN technology.

## 2. Experiment

### 2.1. Sample Preparation

**Figure 1:**
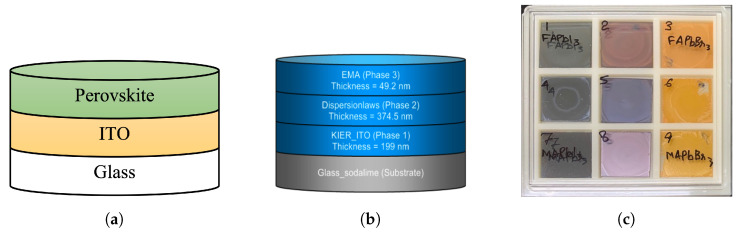
Perovskite material specimen defined by quaternary stoichiometric ratio; (**a**) thin-film laminated structure of specimen; (**b**) optical model structure for ellipsometry; (**c**) Perovskite samples.

As shown in Figure 1a, we used a soda–lime glass substrate uniformly coated with 200 nm of ITO. The thickness of the substrate was 0.8 mm and the thin film stacking area was 25 mm × 25 mm. Perovskite was deposited on the substrate by the spin-coating method, and nine specimens were prepared with three FA and iodine compositions by adjusting the stoichiometric ratio.

#### 2.1.1. Perovskite Precursor Solution Preparation:

The materials used for perovskite precursor solutions preparation include PbI${}_{2}$ (Sigma-Aldrich, St. Louis, MI, USA), PbBr${}_{2}$ (Sigma-Aldrich), CH${}_{3}$NH${}_{3}$I (MAI) (Greatcell Solar), CH${}_{3}$NH${}_{3}$Br (MABr) (Greatcell Solar), CH(NH${}_{2}$)${}_{2}$I (FAI) (Greatcell Solar), CH(NH${}_{2}$)${}_{2}$Br (FABr) (Ossila), dimethyl formamide (DMF) (Sigma-Aldrich), and dimethyl sulfoxide (DMSO) (Sigma-Aldrich). All the chemicals used were anhydrous and were used as received without further purification. A total of nine perovskite precursor solutions were prepared as given in Table 1. Adopting the solution stoichiometry aspects from a perovskite compositional space exploration study by Jacobsson et al. [37], the molar concentration of the [Pb${}^{2+}$] in final solution was kept at a molar ratio of 1.25, which was higher than the accumulative molar concentration of organic salts, i.e., FA and MA. Precisely, the [Pb${}^{2+}$]/([MA] + [FA]) ratio for all precursor solutions was 1.1. The molar concentration for each precursor for each perovskite composition is given in Table 1 for 1mL solvent mix of DMF/DMSO (ratio 1 to 4). All the solutions were prepared in a nitrogen filled-glovebox. After weighing the precursors, the solutions were stirred for 3 h at room temperature inside the glovebox.

#### 2.1.2. Substrate Preparation:

As a substrate, we used a commercially available ITO glass with sheet resistance of about 10 Ohm/sq., 0.7 mm thick soda lime glass, and 200 nm thick indium-doped tin oxide (ITO). The substrates were cut to 25 mm × 25 mm sizes and then ultrasonicated in a bath of deionized water, acetone, and ethyl alcohol for 15 min each to remove any impurities from the ITO surface. The remnant ethanol from the sonication process was dried by nitrogen blowing, followed by further drying in a heat oven at 80 ${}^{\circ }$C for 10 min. The ITO surface is highly hydrophobic; therefore, just before the perovskite deposition, the ITO surface was subjected to an ozone plasma treatment for 15 min at 400 W power. This step ensures the removal of residual organic substances from the ITO surface as well as making it hydrophilic for good coverage of perovskite during the spin-coating step.

#### 2.1.3. Perovskite Deposition:

Next, 100 μL of the prepared perovskite solution was poured and spread onto the ozone-plasma-treated ITO glass. The substrate was rotated at 1000 rpm for 10 s with a 5 s ramp, followed by spinning at 4000 rpm for 30 s with a 2 s ramp. Using a pipette with a pipette tip cut to a relatively larger hole size of 100 μL, anhydrous chlorobenzene (Sigma-Aldrich), an anti-solvent, was delivered uniformly onto the center of the rotating substrate approximately 15 s before the end of the spinning. This step normally introduces change of color of the film due to supersaturation, and after spin-coating, the films were immediately heat-treated at 100 ${}^{\circ }$C for a time duration between 30 and 60 min depending upon the composition used. One exception was the case of the FAPbI${}_{3}$ sample, which was heat treated at 150 ${}^{\circ }$C for 15 min because it needs a higher temperature to form an alpha phase. As the substrate is put on a hot plate, the transformation of the perovskite precursor film to perovskite is visually observable within first few seconds to a minute, depending upon the composition. After heat treatment, the samples were cooled down to room temperature and then sent for further analyses.

#### 2.1.4. Sample Preparation for SEM Analysis:

The samples for scanning electron microscopy (SEM) analysis were prepared according to the procedure described in Fabrication Process section. However, the samples were cut to expose their cross-sections. The samples were mounted onto a SEM sample holder and a thin osmium coating was applied via chemical vapor deposition (CVD) for better visibility and to prevent sample charging during SEM analysis. The SEM analysis was conducted on a NovaNano SEM 450/FEI equipment.

### 2.2. Ellipsometry Analysis

Variable-angle spectroscopic ellipsometry and spectrophotometer measurements were performed on the specimens, as shown in Figure 1c. A phase-modulated spectroscopic ellipsometer (SEMILAB, GES5-E) was used to determine the refractive index and extinction coefficient of the quaternary perovskite material (MA${}_{x}$FA${}_{1-x}$Pb(I${}_{y}$Br${}_{1-y}$)${}_{3}$). The ellipsometer used in the study had a spectrum range of 245–976 nm, beam spot >1.5 mm, and measurement accuracy of sub-$\overset{˚}{A}∼$10 μm. Considering the Brewster angle of the perovskite specimen, the ellipsometry incident beam angle was set to 75${}^{\circ }$. In the ellipsometer measurement process, various characteristics, such as surface roughness, void content, texture, and bulk composition inhomogeneity of the perovskite material, were measured. An optical model using a Semilab spectroscopic ellipsometry analyzer program was established to analyze the characteristics of the specimen obtained from the ellipsometer measurement process. The Bruggeman effective medium approximation (EMA) model was applied to analyze the influence of the irregular texture structure caused by the surface roughness of the perovskite material [38,39]. The EMA model is used very frequently in ellipsometry analysis. If the optical layer is mixed in the thin-film structure, it is approximated by mixing the optical information of the individual components to calculate the optical properties of the layer. In this study, the empty space outside the specimen was defined as a void, and the rough perovskite surface was mixed with the values defined in the bulk dispersion optical layer model. The Bruggeman equation related to the effective refractive index ($n_{eff}$) of a two-component system of the studied material is as follows [40].

(1) $$\nu_{V}\frac{n_{V}-n_{eff}}{n_{V}+\left(d-1\right)n_{eff}}+\nu_{Perov}\frac{n_{Perov}-n_{eff}}{n_{Perov}+\left(d-1\right)n_{eff}}=0$$

where the refractive index of the void is $n_{V}$ and that of perovskite is $n_{Perov}$. The volume fractions of the two phases are represented by $\nu_{V}$ and $\nu_{Perov}$, respectively. The system dimensionality is denoted by *d*, which is 3 for a nanoporous system [41,42]. The total volume fraction is set to 1, and is expressed as $\nu_{V}+\nu_{Perov}=1$. The extinction coefficient can also be expressed as the relational expression in Equation (1).

The modified Forouhi–Bloomer model was applied considering the properties of the non-parabolic energy dependence of the valence and conduction bands of perovskite and ITO, and has the following relation [43,44]:

(2) $$n\left(E\right)=\frac{-\left(B_{0}D+2C_{0}\right)E+2B_{0}F+C_{0}D}{\left(E-D\right)E+F}$$

(3) $$k\left(E\right)=\frac{A+B\left(E-E_{g}\right)+C{\left(E-E_{g}\right)}^{2}}{\left(E-D\right)E+F}$$

(4) $$B_{0}=\frac{B-2CE_{g}+CD}{Q}$$

(5) $$C_{0}=\frac{A-BE_{g}+CE_{g}^{2}-CF}{Q}$$

(6) $$Q=\sqrt{4F-D^{2}}$$

where the law parameters *A*, *B*, *C*, *D*, and *F* have positive non-zero values, and $E_{g}$ represents the optical energy band gap. *A* and *F* have $eV^{2}$ unit values, and *C* is a dimensionless constant.

A new Gaussian dielectric function based on the general form of the Voigt function was applied to define the optical model of amorphous or glass materials [45]. In the new Gaussian optical model, the dielectric constant is defined as the following relational expression:

(7) $$\epsilon_{1}\left(E\right)=\frac{2Amp}{\sqrt{\pi }}\left[D\left(\frac{E+E_{0}}{\sigma }\right)-D\left(\frac{E-E_{0}}{\sigma }\right)\right]$$

(8) $$\epsilon_{2}\left(E\right)=Amp\left[exp\left\{-{\left(\frac{E-E_{0}}{\sigma }\right)}^{2}\right\}-exp\left\{-{\left(\frac{E+E_{0}}{\sigma }\right)}^{2}\right\}\right]$$

where Amp is the oscillator amplitude and $E_{0}$ is the oscillator peak position. The operator defined value is denoted by *D*. The full width at half maximum originating from the Br oscillator broadening is defined as follows.

(9) $$\sigma =\frac{Br}{2\sqrt{ln2}}$$

In this study, a Drude model was used to simulate the conduction of quasi-free electrons in metals or semiconductor materials [38].

(10) $$\epsilon_{1}\left(E\right)=-\frac{{\left(\frac{E_{P}}{E}\right)}^{2}}{1+{\left(\frac{E_{\Gamma }}{E}\right)}^{2}}$$

(11) $$\epsilon_{2}\left(E\right)=\frac{E_{P}}{E}\frac{{\left(\frac{E_{\Gamma }}{E}\right)}^{2}}{1+{\left(\frac{E_{\Gamma }}{E}\right)}^{2}}$$

where $E_{P}$ is the plasma energy and $E_{\Gamma }$ is the broadening related to the scattering frequency.

The optical model structure of the ellipsometer analysis consists of four layers: glass substrate, ITO phase, perovskite phase, and EMA phase, as shown in Figure 1b. The glass substrate was defined as having infinite thickness and was used by applying the well-known nk value of soda-lime glass, and an optical model was designed using the dispersion law for the deposited phase. For the second layer comprising the ITO phase, the Drude and modified Forouhi–Bloomer models were applied to construct the optical model. The perovskite phase, modified Forouhi–Bloomer model, and Gauss model were applied to the third layer of the structure. Finally, to reflect the void effect caused by the surface roughness of the perovskite material, the EMA phase was applied as the fourth layer of the structure.

### 2.3. Hyperregression Analysis

**Figure 2:**
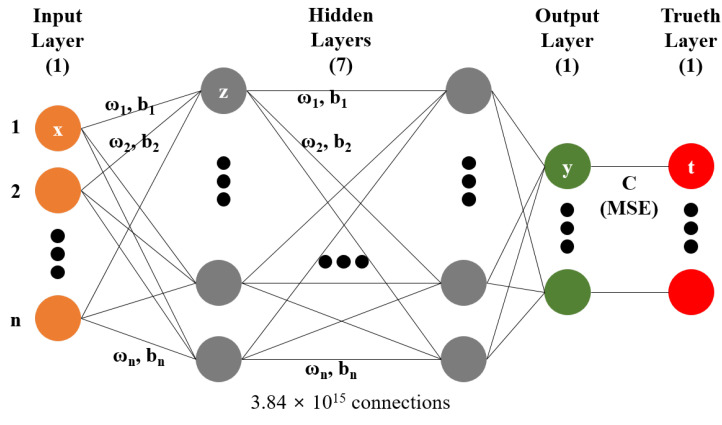
Schematic of fully coupled artificial neural network model.

As shown in Figure 2, the construction of an ANN for performing hyperregression analysis can be defined as follows:

(12) $$z_{1}=\left(\omega_{1}x_{1}+\cdots +\omega_{n}x_{n}+b\right)$$

where *x* and *z* denote the neurons of the input and hidden layers, respectively. The weight of the input signal is expressed as ω, and the neuron threshold, which determines whether the neural signal is output, is called the bias and is denoted as *b*. A rectified linear unit (ReLU) is used as the activation function to generate the output layer for the input layer, and is expressed as follows:

(13) $$a\left(x\right)=\left\{\begin{array}{ccc} 0 & for & x<0\\ x & for & x\geq 0 \end{array}\right.$$

(14) $$y_{1}=a\left(z_{1}\right)$$

(15) $$C_{T},MSE=\frac{1}{n}\sum_{i=1}^{n}{\left(t_{n}-y_{n}\right)}^{2}$$

where $a\left(x\right)$ represents the ReLU algorithm. The notation *y* denotes the neurons of the output layer and *t* denotes the neurons of the ground truth layer. $C_{T}$ is the cost function of the mean squared error (MSE) algorithm. Artificial neural network learning is a process of minimizing the cost function by adjusting the bias and weights that respond to the neurons.

The process of minimizing the cost function is called optimization, and the process of continuously calculating and updating the displacement with respect to the slope of the bias and weight for optimization is called the gradient descent. The minute displacement for the weight and bias of the input neuron using the gradient descent method can be expressed as follows:

(16) $$\Delta \omega_{1}=-\eta \frac{\partial f}{\partial \omega_{1}},\Delta b_{1}=-\eta \frac{\partial f}{\partial b_{1}}$$

(17) $$\left(\Delta \omega_{1},\cdots ,\Delta \omega_{n},\Delta b_{1},\cdots ,\Delta b_{n}\right)=-\eta \left(\frac{\partial f}{\partial \omega_{1}},\cdots ,\frac{\partial f}{\partial \omega_{n}},\frac{\partial f}{\partial b_{1}},\cdots ,\frac{\partial f}{\partial b_{n}}\right)$$

where η is the learning rate of the deep learning process, and fast learning is possible at a high learning rate; however, it is necessary to define an appropriate value, as accuracy is low at very high learning rates.

As the number of variables for deep learning increases, the complexity of the gradient descent method increases, and the partial differential calculation increases exponentially; therefore, it is difficult to quickly verify the result because the amount of calculation increases. To solve this problem, the minimum cost function can be derived using the error backpropagation algorithm. The error backpropagation method defines the slope of the cost function for each neuron as the error δ. By defining the slope of the cost function with respect to the weight and bias as the equation for the error δ, the partial differential calculation of the gradient descent is omitted. In the error backpropagation process, the output layer is calculated by randomly configuring the weight and bias matrix inside the ANN, and the cost function is constructed using the MSE algorithm by comparing it with the verification value. In the generated cost function, an error δ matrix inside the ANN is constructed for each neuron, and the slope of the cost function with respect to the weight and bias is calculated. The initial weights and biases are updated to the subsequent values using the gradient descent method by calculating the differential values of the initial weights and bias matrices to which the learning rate is applied. The process of calculating the output layer and updating the cost function is repeated using the updated weights and biases.

As described earlier, the error backpropagation method can be defined as a repetition of the error minimization process originating from the difference between the true and predicted values with various variables, which is, in principle, the same as statistical regression analysis. The regression analysis shows the linear relationship of discrete data expressed using two variables; for a complex data distribution, the analysis is performed using a polynomial regression model. Here, when there are fewer than three variable terms, the regression can be conveniently analyzed, but when the regression structure has three or more dimensions, we cannot express it using a visual graph, and it is difficult to perform regression analysis on the data.

In general, when visualizing the changes in temperature with time, two variable axes can be set in the graph, and a regression analysis of two-variable data can be performed. Here, the temperature according to the change in time and atmospheric pressure can be visualized, as there are three variable axes in the graph, and linear regression analysis is possible. However, when there are four variable axes in the graph, the temperature according to the change in time, atmospheric pressure, and chemical composition cannot be visualized using a general method; thus, regression analysis is difficult. The change in optical properties according to the stoichiometric ratio of the quaternary compound to be investigated in this study has many variables; hence, it can be predicted by visualizing n-dimensional data and performing regression analysis. However, because regression analysis is not possible with the current algebraic method, a new method for regression analysis is required. As shown in Figure 2, the input item in the machine learning algorithm can be treated as a single variable, and when it increases, the terms of the input layer in the ANN increase. Therefore, in machine learning, regression analysis is theoretically possible by defining infinite variables. The regression curve formed using the artificial intelligence technology increases exponentially according to the number of variables, and the shape of the curve is such that it cannot be expressed with human cognitive ability. In this study, using the artificial intelligence technology to perform regression analysis on data where three or more variables act is defined as hyperregression analysis.

In general, regression analysis through artificial intelligence methods can often be used to explain data classification and trends. In the case of the optical properties to be analyzed in this study, because the refractive index and extinction coefficient according to the change in wavelength comprise one unit of data, artificial intelligence specialized in pattern analysis is often used to interpret them. In the case of such a CNN-based pattern analysis, it is difficult to secure a result that is not learned by the classification standard because it is focused on the classification and discrimination of the shape of the irregular pattern. To predict the optical properties of perovskite by machine learning, it is necessary to construct big data based on the actual data and to learn the information stored in these data. The first problem is configuring big data to perform machine learning. The process of machine learning generally requires big data with at least 1000 data units. Nine specimens were prepared in this study; hence, there were nine patterns of the refractive index and extinction coefficient, constituting a very small quantity of data. It is difficult to use these data to increase the efficiency of the experiments because a large amount of data is required to build big data based on patterns.

**Figure 3:**
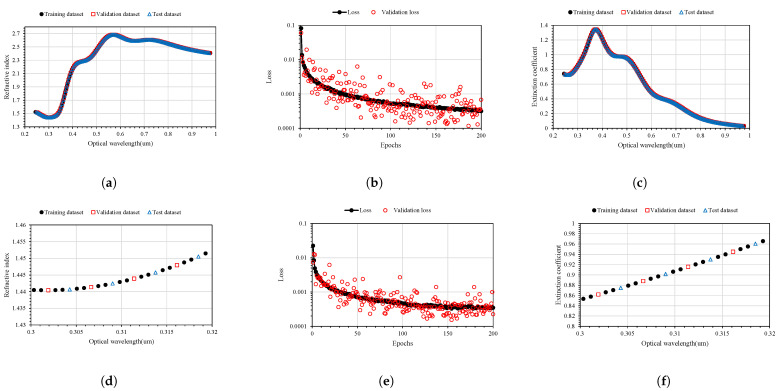
AI training, validation, and test data sets of measured optical properties on FAPbI${}_{3}$ and learning loss progress with epochs: (**a**) whole refractive index data set of FAPbI${}_{3}$, (**b**) AI learning progress on refractive index of quaternary perovskite with loss and validation loss, (**c**) whole extinction coefficient data set of FAPbI${}_{3}$, (**d**) extension refractive index data set of FAPbI${}_{3}$, (**e**) AI learning progress on extinction coefficient of quaternary perovskite with loss and validation loss, (**f**) extension extinction coefficient data set of FAPbI${}_{3}$.

A key approach to build big data using a minimal set of specimens is to change the general perspective on the shape of the data. Instead of representing the entire refractive index pattern according to the wavelength as a single unit of data, if the refractive index value for each wavelength is represented as a unit of data, it is possible to construct big data by accumulating a large amount of data despite the small number of specimens. In conclusion, the parameters for machine learning are defined as the stoichiometric ratio of MA, FA, I${}_{3}$, and Br${}_{3}$ and the wavelength as input variables, and the corresponding refractive index or extinction coefficient as the output variable. Through the ellipsometry analysis used in this study, it is possible to analyze 928 refractive indices and extinction coefficients for the wavelengths of 244–978 nm at intervals of 1 nm per specimen. By considering only nine points to produce the minimum number of specimens within the range of the perovskite composition, as shown in Table 2, 6612 big data were configured to perform machine learning for the refractive index and extinction coefficient. A data set was constructed for training and validation. The configured data set was classified into a training data set of 4408 data points(66.6%), a validation data set of 1102 data points (16.7%), and a test data set of 1102 data points (16.7%). The data set distribution of FAPbI${}_{3}$ material was defined as shown in Figure 3. As can be seen in Figure 3a,c, the optical characteristic pattern of the FAPbI${}_{3}$ material can be confirmed regardless of the type of data set. As can be seen in Figure 3d,f, the distribution of the specific data set was defined by selecting the data at intervals of 6 nm in the wavelength band within the AI learning data range and defining the validation and test data sets.

Thus, we conducted machine learning using the nine results corresponding to the ellipsometry analysis of the fabricated perovskite samples, and the weight and bias matrix with the minimized cost function were defined by learning of the ANN model. The refractive index and extinction coefficient corresponding to a wavelength can be confirmed by inputting the values of the stoichiometric ratio and wavelength used in the experiment to the learned artificial intelligence model. If these results are repeated using the artificial intelligence model for wavelengths of 300–900 nm at intervals of 1 nm, a pattern graph that is almost identical to the optical information analyzed through the specimen can be obtained. This technique of performing machine learning is called hyperregression analysis, and in addition to spectral prediction, interpolation approximations of various parts can be easily derived. Deviating from the concept of pattern-type recognition for the entire spectrum, the proposed approach achieves results similar to those of the regression analysis of the tendency of the refractive index and extinction coefficient corresponding to a single wavelength to change with the increase in FA or Br${}_{3}$.

We used TensorFlow (ver.2.0.0) to perform machine learning, and KERAS (ver.2.2.4) was applied as the backend module. KERAS was used to construct the ANN, which adopted a sequential structure. As shown in Figure 2, seven hidden layers were used, and the number of neurons was configured differently for each layer. ReLU was used for the activation function of each layer. Multi-GPU calculation was performed to train the ANN. To perform deep learning, the optimizer applied the root mean square propagation algorithm modified from stochastic gradient descent. The MSE algorithm was used to perform a regression analysis for the loss function of machine learning.

## 3. Results

### 3.1. Ellipsometry Measurement

**Figure 4:**
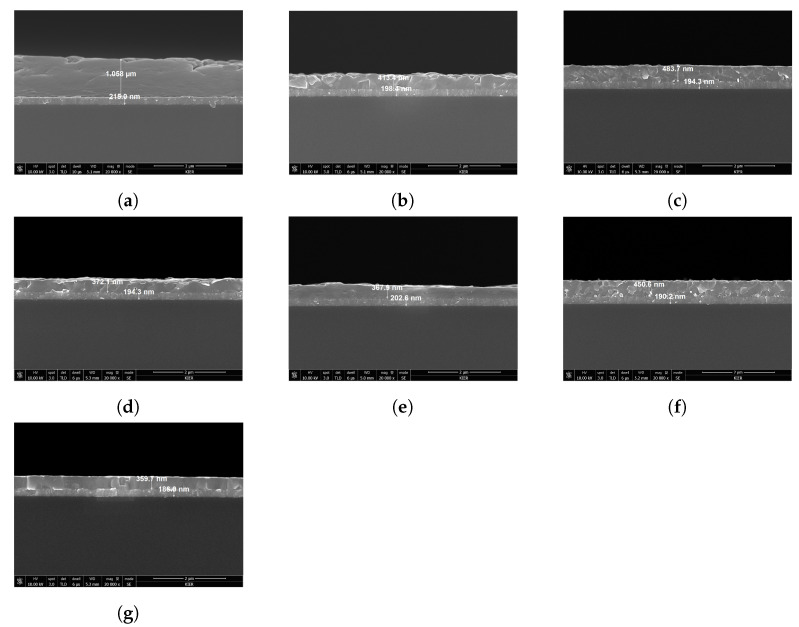
Analysis of the cross-sectional structure of quaternary perovskite specimens using SEM: (**a**) FAPbI${}_{3}$, (**b**) FAPbBr${}_{3}$, (**c**) FA${}_{0.5}$MA${}_{0.5}$PbI${}_{3}$, (**d**) FA${}_{0.5}$MA${}_{0.5}$PbI${}_{1.5}$Br${}_{1.5}$, (**e**) FA${}_{0.5}$MA${}_{0.5}$Br${}_{3}$, (**f**) MAPbI${}_{3}$, (**g**) MAPbBr${}_{3}$.

As shown in Figure 4, the cross-section of the specimen was measured using SEM to confirm the thicknesses of ITO and perovskite to be used in the optical model for ellipsometry analysis. Two samples, FAPbBr${}_{1.5}$I${}_{1.5}$ and MAPbBr${}_{1.5}$I${}_{1.5}$, were damaged during the preparation for SEM analysis. The bottom layer was a soda-lime glass substrate, and it can be seen in Figure 4 that an ITO layer with a thickness of approximately 200 nm was positioned on it. The thickness of the ITO layer was confirmed through the cross-sectional measurement of the specimen using SEM, and it was defined accordingly in the ellipsometry optical model.

**Figure 5:**
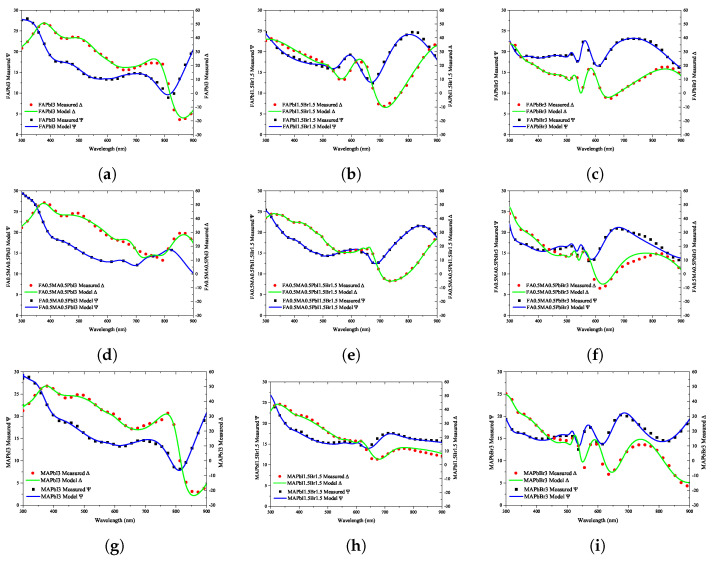
Measurement results using ellipsometry analysis of quaternary perovskite materials and optical simulation results: (**a**) FAPbI${}_{3}$, (**b**) FAPbI${}_{1.5}$PbBr${}_{1.5}$, (**c**) FAPbBr${}_{3}$, (**d**) FA${}_{0.5}$MA${}_{0.5}$PbI${}_{3}$, (**e**) FA${}_{0.5}$MA${}_{0.5}$PbI${}_{1.5}$Br${}_{1.5}$, (**f**) FA${}_{0.5}$MA${}_{0.5}$Br${}_{3}$, (**g**) MAPbI${}_{3}$, (**h**) MAPbI${}_{1.5}$Br${}_{1.5}$, (**i**) MAPbBr${}_{3}$.

The fabricated perovskite specimens were measured through ellipsometry, and the measured results are summarized in the ψ and Δ shapes in Figure 5. The measured results included all information of the fabricated perovskite specimen, namely, the surface roughness, perovskite thickness, and optical properties of ITO and the glass substrate. Regarding the composition of nine perovskites, an optical model reflecting all the information was established; the results were fitted to the experimental results, and good agreement was observed over the entire wavelength range.

**Figure 6:**
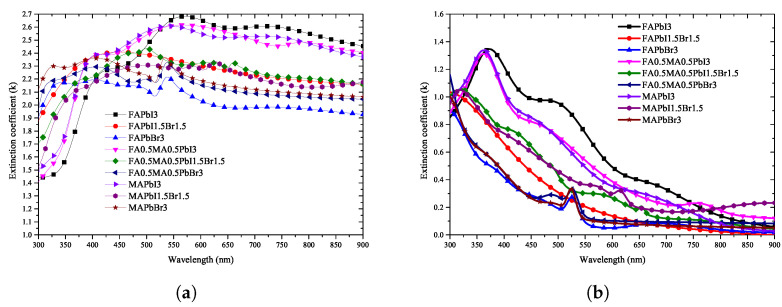
Analysis of optical properties through ellipsometry analysis of quaternary perovskite materials: (**a**) refractive index, (**b**) extinction coefficient.

As shown in Figure 6, the refractive index and extinction coefficient of the perovskite material were extracted using ellipsometry optical analysis. The optical properties of only the deposited perovskite were accurately extracted by excluding the optical properties of the ITO and glass substrates of the manufactured specimen using the optical model defined above. The extracted refractive index and extinction coefficient were divided into the compositional ratios according to the stoichiometric ratio.

**Figure 7:**
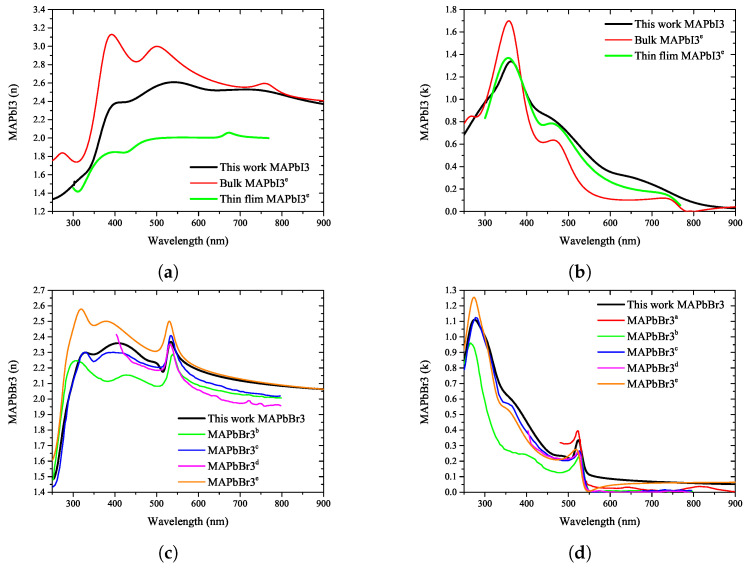
The optical property measurement results of the ellipsometry analysis of quaternary perovskite materials in this study were compared with the reference data from previously reported papers ${}^{a-e}$ [37,46,47,48,49]. (**a**) refractive index of MAPbI${}_{3}$, (**b**) extinction coefficient of MAPbI${}_{3}$, (**c**) refractive index of MAPbBr${}_{3}$, (**d**) extinction coefficient of MAPbBr${}_{3}$.

To confirm the reliability of the optical properties of the perovskite extracted in this study, they were compared with the optical properties published in a previous study. Figure 7 shows the comparison between the literature data and measurement results for two compositions, MAPbI${}_{3}$ and MAPbBr${}_{3}$, which are typically used in quaternary perovskite.

### 3.2. AI Training Results

**Figure 8:**
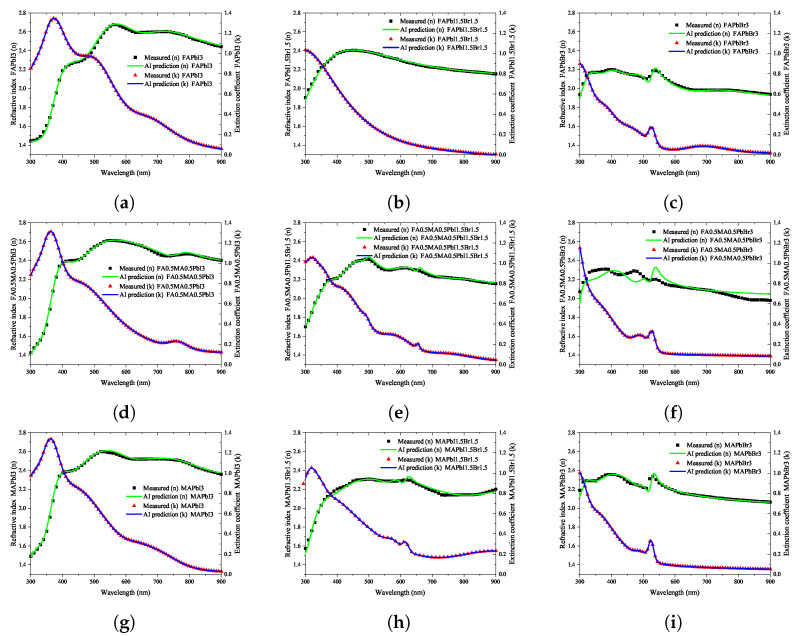
Refractive index and extinction coefficient through ellipsometry analysis of the quaternary perovskite material and comparison of the results of hyperregression analysis using artificial intelligence models: (**a**) FAPbI${}_{3}$, (**b**) FAPbI${}_{1.5}$PbBr${}_{1.5}$, (**c**) FAPbBr${}_{3}$, (**d**) FA${}_{0.5}$MA${}_{0.5}$PbI${}_{3}$, (**e**) FA${}_{0.5}$MA${}_{0.5}$PbI${}_{1.5}$Br${}_{1.5}$, (**f**) FA${}_{0.5}$MA${}_{0.5}$Br${}_{3}$, (**g**) MAPbI${}_{3}$, (**h**) MAPbI${}_{1.5}$Br${}_{1.5}$, (**i**) MAPbBr${}_{3}$.

As shown in Figure 3b, AI learning of 200 epochs was performed using the defined data set. In the learned refractive index prediction AI, the loss of the training data set and the validation loss using the validation data set were 3.1652 × 10${}^{-4}$ and 6.7654 × 10${}^{-4}$, respectively. As can be seen in Figure 3e, in the case of the extinction coefficient prediction AI, the loss of the training data set and the validation loss using the validation data set were 3.4941 × 10${}^{-4}$ and 2.2626 × 10${}^{-4}$, respectively. As summarized in Figure 8, the prediction results obtained with artificial intelligence learned using the big data constructed in this study were compared with the experimental results for changes in the composition. To predict the optical information related to the change in stoichiometric ratio by using artificial intelligence, a continuous input data matrix was created in the format of the input neuron data shown in Table 2 and extracted for the entire spectrum.

**Figure 9:**
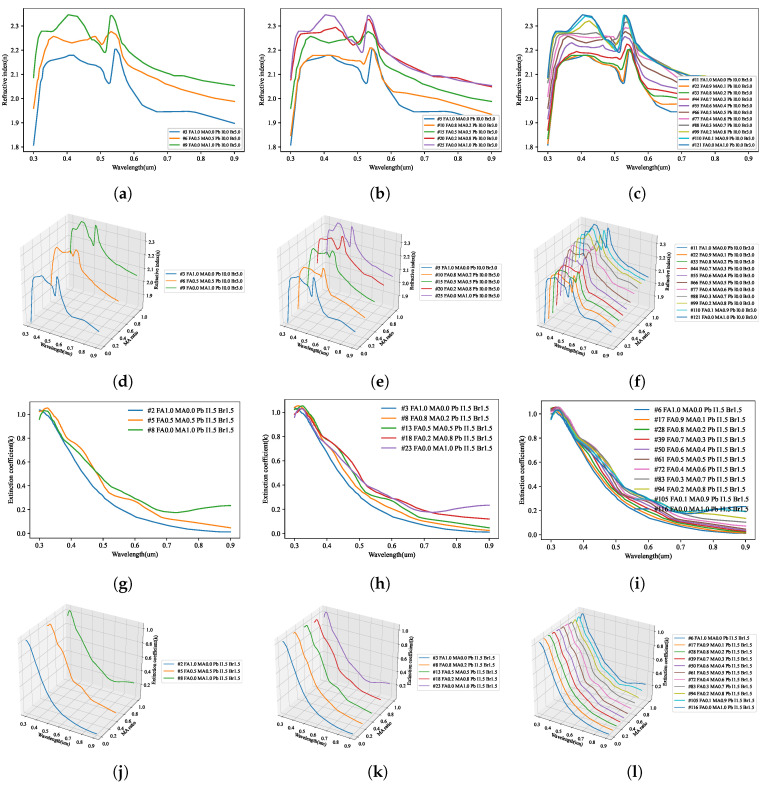
Prediction result of refractive index and the extinction coefficient spectrum pattern according to the stoichiometric ratio in the direction of increasing MA composition of quaternary perovskite using hyperregression analysis with artificial intelligence: (**a**,**d**) refractive index of AI expectation for experimental optical data on FA${}_{1-x}$MA${}_{x}$PbBr${}_{3}$ with 2D and 3D plot, (**b**,**e**) refractive index of hyperregression result on interpolation between experimental optical data of FA${}_{1-x}$MA${}_{x}$PbBr${}_{3}$ with 2D and 3D plot, (**c**,**f**) refractive index of finer hyperregression result on interpolation between experimental optical data of FA${}_{1-x}$MA${}_{x}$PbBr${}_{3}$ with 2D and 3D plot, (**g**,**j**) extinction coefficient of AI expectation for experimental optical data on FA${}_{1-x}$MA${}_{x}$PbI${}_{1.5}$Br${}_{1.5}$ with 2D and 3D plot, (**h**,**k**) extinction coefficient of hyperregression result on interpolation between experimental optical data of FA${}_{1-x}$MA${}_{x}$PbI${}_{1.5}$Br${}_{1.5}$ with 2D and 3D plot, (**i**,**l**) extinction coefficient of finer hyperregression result on interpolation between experimental optical data of FA${}_{1-x}$MA${}_{x}$PbI${}_{1.5}$Br${}_{1.5}$ with 2D and 3D plot.

As shown in Figure 9, the optical properties of the quaternary perovskite were predicted using the learned AI. As confirmed in Figure 8, the results of the refractive index spectrum predicted through AI are summarized in Figure 9c. When confirmed in three-dimensional space, it appears in the form of Figure 9d, and a constant spectrum deformation pattern is observed. The results shown in Figure 9e were obtained using the learned AI to estimate the interpolation value between the measured optical properties. We obtained the refractive index information predicted from the compositions of FA${}_{0.8}$ MA${}_{0.2}$ PbBr${}_{3}$ and FA${}_{0.2}$ MA${}_{0.8}$ PbBr${}_{3}$, which were not produced in this study. Thus, the interpolation value between the experimentally measured values was more finely defined to obtain dense refractive index information, as shown in Figure 9f.

**Figure 10:**
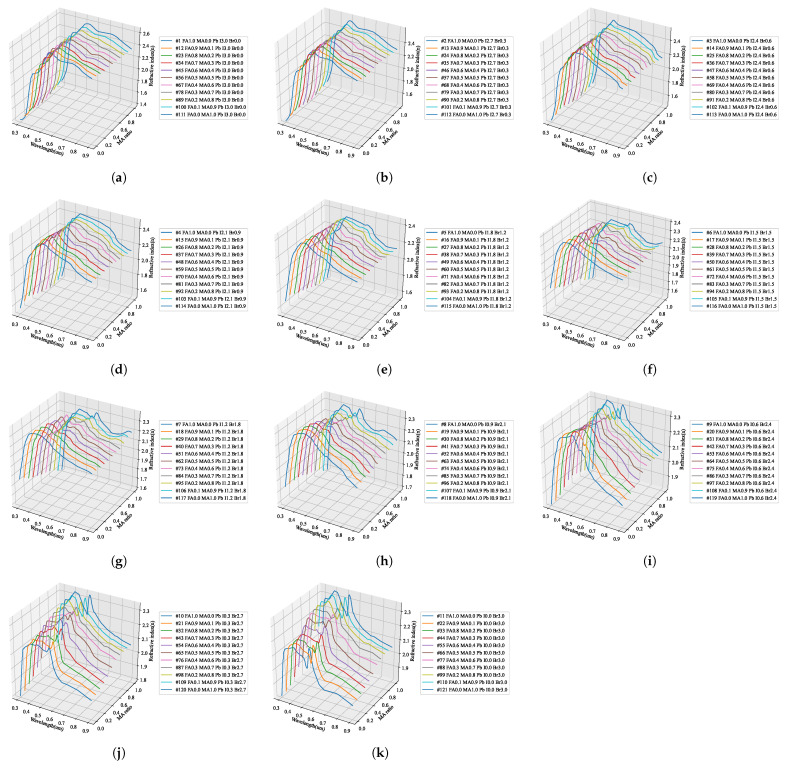
Prediction result of the refractive index spectrum pattern according to the stoichiometric ratio in the direction of increasing MA composition of quaternary perovskite using hyperregression analysis with artificial intelligence: (**a**) FA${}_{1-x}$MA${}_{x}$PbI${}_{3}$Br${}_{0}$, (**b**) FA${}_{1-x}$MA${}_{x}$PbI${}_{2.7}$Br${}_{0.3}$, (**c**) FA${}_{1-x}$MA${}_{x}$PbI${}_{2.4}$Br${}_{0.6}$, (**d**) FA${}_{1-x}$MA${}_{x}$PbI${}_{2.1}$Br${}_{0.9}$, (**e**) FA${}_{1-x}$MA${}_{x}$PbI${}_{1.8}$Br${}_{1.2}$, (**f**) FA${}_{1-x}$MA${}_{x}$PbI${}_{1.5}$Br${}_{1.5}$, (**g**) FA${}_{1-x}$MA${}_{x}$PbI${}_{1.2}$Br${}_{1.8}$, (**h**) FA${}_{1-x}$MA${}_{x}$PbI${}_{0.9}$Br${}_{2.1}$, (**i**) FA${}_{1-x}$MA${}_{x}$PbI${}_{0.6}$Br${}_{2.4}$, (**j**) FA${}_{1-x}$MA${}_{x}$PbI${}_{0.3}$Br${}_{2.7}$, (**k**) FA${}_{1-x}$MA${}_{x}$PbI${}_{0}$Br${}_{3}$.

**Figure 11:**
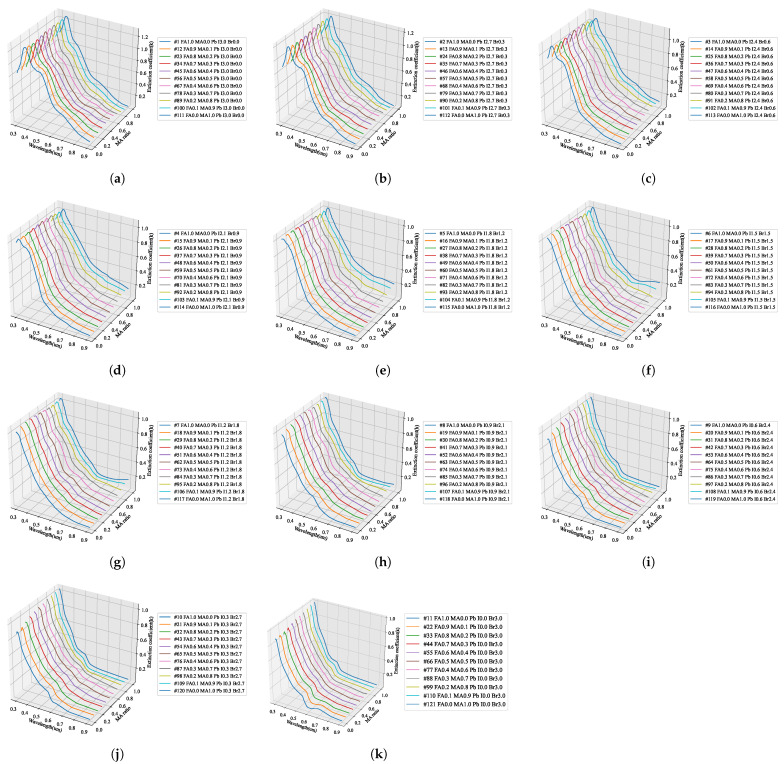
Prediction result of the extinction coefficient spectrum pattern according to the stoichiometric ratio in the direction of increasing MA composition of quaternary perovskite using hyperregression analysis with artificial intelligence: (**a**) FA${}_{1-x}$MA${}_{x}$PbI${}_{3}$Br${}_{0}$, (**b**) FA${}_{1-x}$MA${}_{x}$PbI${}_{2.7}$Br${}_{0.3}$, (**c**) FA${}_{1-x}$MA${}_{x}$PbI${}_{2.4}$Br${}_{0.6}$, (**d**) FA${}_{1-x}$MA${}_{x}$PbI${}_{2.1}$Br${}_{0.9}$, (**e**) FA${}_{1-x}$MA${}_{x}$PbI${}_{1.8}$Br${}_{1.2}$, (**f**) FA${}_{1-x}$MA${}_{x}$PbI${}_{1.5}$Br${}_{1.5}$, (**g**) FA${}_{1-x}$MA${}_{x}$PbI${}_{1.2}$Br${}_{1.8}$, (**h**) FA${}_{1-x}$MA${}_{x}$PbI${}_{0.9}$Br${}_{2.1}$, (**i**) FA${}_{1-x}$MA${}_{x}$PbI${}_{0.6}$Br${}_{2.4}$, (**j**) FA${}_{1-x}$MA${}_{x}$PbI${}_{0.3}$Br${}_{2.7}$, (**k**) FA${}_{1-x}$MA${}_{x}$PbI${}_{0}$Br${}_{3}$.

**Figure 12:**
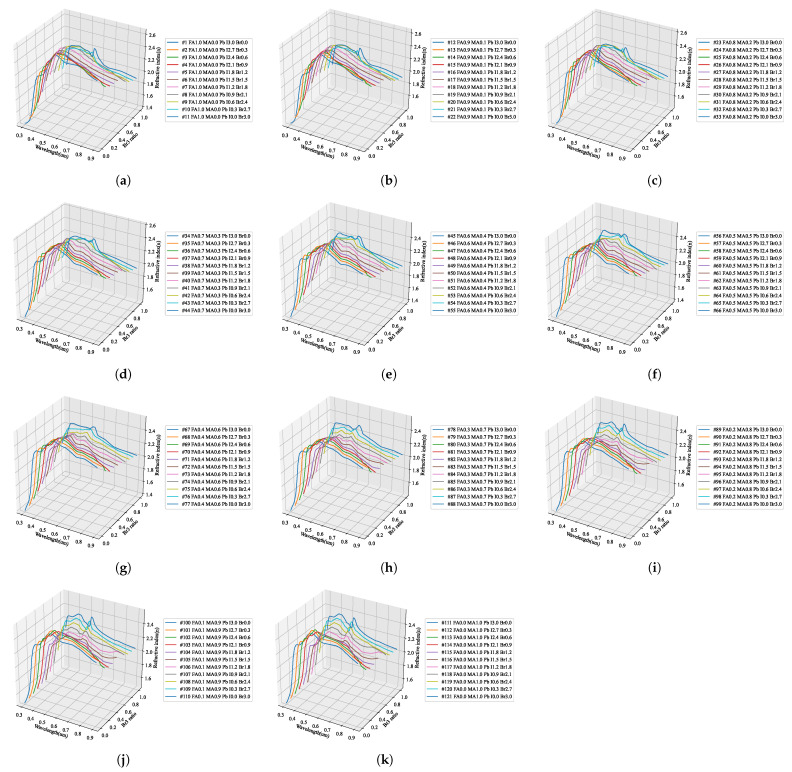
Prediction result of the refractive index spectrum pattern according to the stoichiometric ratio in the direction of increasing Br${}_{3}$ composition of quaternary perovskite using hyperregression analysis with artificial intelligence: (**a**) FA${}_{1}$MA${}_{0}$PbI${}_{3-y}$Br${}_{y}$, (**b**) FA${}_{0.9}$MA${}_{0.1}$PbI${}_{3-y}$Br${}_{y}$, (**c**) FA${}_{0.8}$MA${}_{0.2}$PbI${}_{3-y}$Br${}_{y}$, (**d**) FA${}_{0.7}$MA${}_{0.3}$PbI${}_{3-y}$Br${}_{y}$, (**e**) FA${}_{0.6}$MA${}_{0.4}$PbI${}_{3-y}$Br${}_{y}$, (**f**) FA${}_{0.5}$MA${}_{0.5}$PbI${}_{3-y}$Br${}_{y}$, (**g**) FA${}_{0.4}$MA${}_{0.6}$PbI${}_{3-y}$Br${}_{y}$, (**h**) FA${}_{0.3}$MA${}_{0.7}$PbI${}_{3-y}$Br${}_{y}$, (**i**) FA${}_{0.2}$MA${}_{0.8}$PbI${}_{3-y}$Br${}_{y}$, (**j**) FA${}_{0.1}$MA${}_{0.9}$PbI${}_{3-y}$Br${}_{y}$, (**k**) FA${}_{0}$MA${}_{1}$PbI${}_{3-y}$Br${}_{y}$.

**Figure 13:**
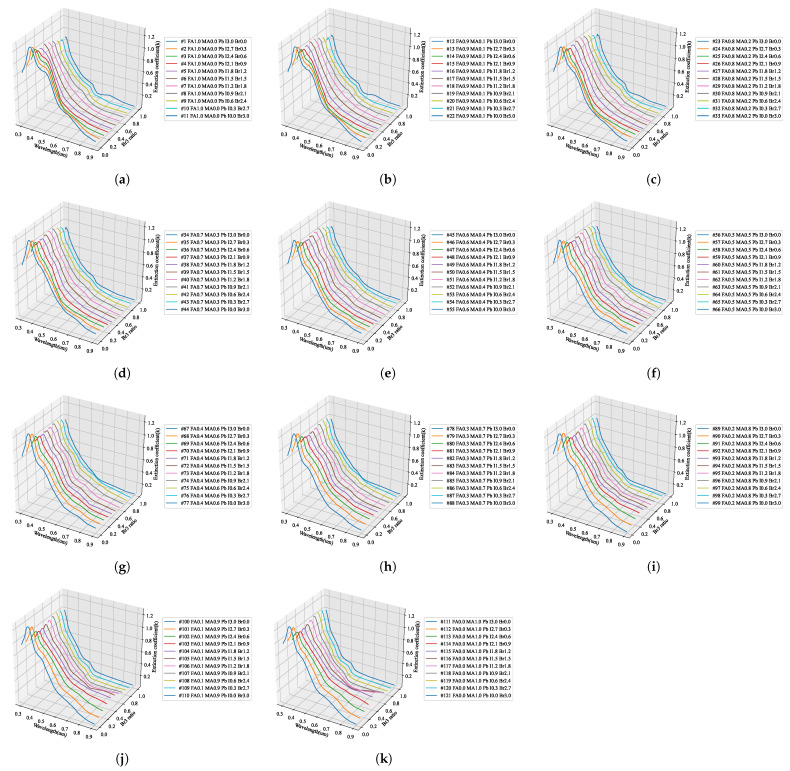
Prediction result of the extinction coefficient spectrum pattern according to the stoichiometric ratio in the direction of increasing Br${}_{3}$ composition of quaternary perovskite using hyperregression analysis with artificial intelligence: (**a**) FA${}_{1}$MA${}_{0}$PbI${}_{3-y}$Br${}_{y}$, (**b**) FA${}_{0.9}$MA${}_{0.1}$PbI${}_{3-y}$Br${}_{y}$, (**c**) FA${}_{0.8}$MA${}_{0.2}$PbI${}_{3-y}$Br${}_{y}$, (**d**) FA${}_{0.7}$MA${}_{0.3}$PbI${}_{3-y}$Br${}_{y}$, (**e**) FA${}_{0.6}$MA${}_{0.4}$PbI${}_{3-y}$Br${}_{y}$, (**f**) FA${}_{0.5}$MA${}_{0.5}$PbI${}_{3-y}$Br${}_{y}$, (**g**) FA${}_{0.4}$MA${}_{0.6}$PbI${}_{3-y}$Br${}_{y}$, (**h**) FA${}_{0.3}$MA${}_{0.7}$PbI${}_{3-y}$Br${}_{y}$, (**i**) FA${}_{0.2}$MA${}_{0.8}$PbI${}_{3-y}$Br${}_{y}$, (**j**) FA${}_{0.1}$MA${}_{0.9}$PbI${}_{3-y}$Br${}_{y}$, (**k**) FA${}_{0}$MA${}_{1}$PbI${}_{3-y}$Br${}_{y}$.

We predicted the fine optical information in the same way for the remaining extinction coefficients. Within the stoichiometric composition range of the perovskite, the optical properties, including the composition without actual specimens, were predicted and are summarized in Figure 10, Figure 11, Figure 12 and Figure 13. By using the learned artificial intelligence model, 11 conditions were defined according to the addition ratio of MA and Br${}_{3}$, and the refractive index and extinction coefficient were predicted for a total of 121 compositions. The graphs that summarize the predicted results according to the MA addition ratio are shown in Figure 10 and Figure 11, and 11 graphs were obtained according to the increase in the composition of Br${}_{3}$. The prediction results by adding Br${}_{3}$ are presented in Figure 12 and Figure 13.

## 4. Discussion

### 4.1. Ellipsometry Measurement

As shown in Figure 4, the perovskite material was deposited on the uppermost layer; a thickness distribution of 350–1058 nm was confirmed depending on the specimen preparation conditions. It can be seen that the thickness of the perovskite layer changes according to the stoichiometric ratio of the specimen and the crystallization conditions, and the shape and surface roughness of the produced crystals vary. As can be confirmed from these measurement results, it is difficult to produce perovskite of the same thickness even when the specimen is manufactured under the same conditions. Further, it can be seen that the state of the surface varies greatly depending on the environmental factors. Other researchers attempting to reproduce the same conditions to fabricate the specimens may not achieve the same thickness of the perovskite and shape of the crystals. In summary, because it is difficult to consistently achieve the same stoichiometric ratio of the perovskite, it is necessary to create an artificial intelligence model to predict changes in the physical properties according to the change in the composition of the perovskite within the limits of the chemical composition used.

Figure 5 shows the ellipsometry results of the optical properties of the fabricated perovskite specimens. Because the specimens had the same structure and properties except for the perovskite, the changes in the patterns of ψ and Δ depending on the wavelength can be compared according to the change in the stoichiometric ratio. From Figure 5a–c, it can be seen that the patterns of ψ and Δ change dramatically as the addition of Br${}_{3}$ increases. On the contrary, it can be seen that the patterns of ψ and Δ do not change significantly as the added quantity of MA increases. Moreover, it is confirmed from Figure 5a–g that the peak positions appearing in the 400, 500, and 750 nm wavelengths were maintained similarly. As can be seen from the color of the fabricated perovskite shown in Figure 1c, this change is similar; although the addition of FA does not change the color noticeably, a significant color change is observed because of Br${}_{3}$. Based on the above trends, it was verified that the ellipsometry measurement results achieved in this study well reflect the optical properties of the fabricated specimens.

Because the results measured using ellipsometry include the information of all thin-film layers shown in Figure 1a, an optical model of the form shown in Figure 1b was constructed and simulated for comparison with the calculation results. Through the optical model, all the information generated from the perovskite specimen was analyzed; the information of the ITO film could be separated from that of the glass substrate so that the physical properties of the perovskite bulk thin-film layer could be accurately calculated. As shown in Figure 5, the information of the perovskite specimen measured using ellipsometry and the simulation result using the optical model were compared, and consistent results were obtained for all perovskite specimens. The refractive index of the perovskite extracted using the ellipsometry optical model is shown in Figure 6a; it can be seen that the refractive index is in the range of 1.4–2.7 for wavelengths between 300–900 nm. Figure 6a also shows that the extinction coefficient of perovskite is 0.0–1.4 for wavelengths between 300–900 nm.

Because the refractive index is maintained uniformly at all wavelengths, reflection from the thin film may occur in the entire range of visible light during the process of manufacturing a perovskite solar cell. To minimize this reflection, it is necessary to adjust the stacking order and thickness in consideration of the refractive index of the thin film in contact with the perovskite. Because the optical properties of the thin-film layer can be calculated using the transfer matrix method, knowledge of the correct refractive index for each wavelength can help design the thin-film structure of the perovskite solar cell. In contrast, it can be seen that the extinction coefficient of perovskite has a high value in the 300–500 nm region, which is consistent with the generally known absorption characteristics of perovskite in the short-wavelength range. The size of the solar spectrum absorbed by the upper cell in the multi-junction solar cell can be predicted because the peak position at which light absorption is maximized is located in the short-wavelength band. Because the refractive index and extinction coefficient change in an irregular pattern according to the change in stoichiometric composition of the quaternary perovskite material, analysis of the optical characteristics is necessary for all compositions to design a structure with optimal optical characteristics according to the composition.

To confirm the reliability of the ellipsometry analysis of the perovskite analyzed in this study, Figure 7 compares the optical properties such as the refractive index and extinction coefficient with those reported in the literature. As shown in Figure 7a, the results measured in this study are between those reported for bulk and thin-film perovskite materials in the literature. Thus, it can be seen that the value of the refractive index changes significantly with change in the thickness of the perovskite. Compared with the results of this study, the characteristic peaks generated in the 400, 500, and 750 nm wavelength bands were found to be similar to those in the referenced literature. As shown in Figure 7b, the results of the extinction coefficient analysis are also judged comparable to those in literature, showing a smaller difference than the refractive index. In addition, it was confirmed that the characteristic absorption peak was observed in the wavelength range around 380 nm, and the pattern of the extinction coefficient decreased with increasing wavelength.

As shown in Figure 7c, in the case of MAPbBr${}_{3}$, the results observed in this study and those reported in the referenced studies are very similar. In particular, characteristic peaks in the wavelength range of 550 nm can be confirmed in all measurement results, and it can be verified that the shapes of the peaks found at 300 and 400 nm are the same. Figure 7d shows that the measurement result of the extinction coefficient has a pattern similar to that in the referenced literature. The characteristic absorption peaks in the wavelength range of 300 and 550 nm observed in the results of this study were found to have the same patterns as those in the referenced papers. The patterns depict a similar decrease in the absorption rate with increasing wavelength.

A comparison of the results with the referenced literature, depicted in Figure 7, shows that the results of perovskite materials with the same stoichiometric ratio are comparable, but not entirely consistent, because of the difference in the environmental conditions during fabrication. Because it is impossible to accurately reproduce the research results owing to the difference in the optical properties, the construction of an artificial intelligence model that can predict the change in the optical properties according to the stoichiometric ratio applied in the research environment of different researchers is considered absolutely necessary.

**Figure 14:**
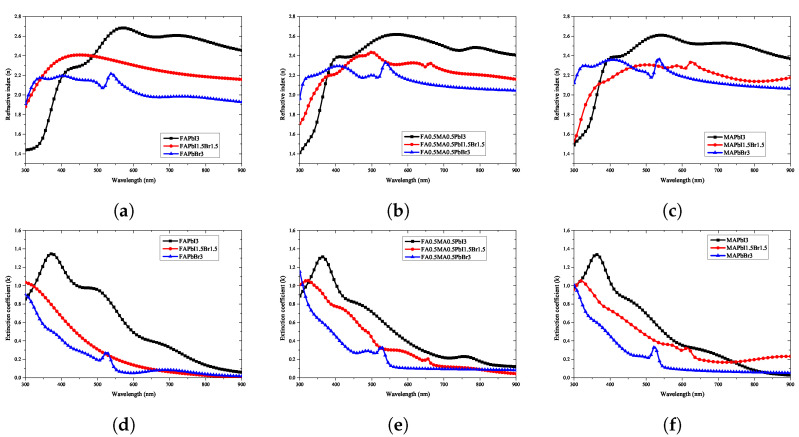
Results of optical properties with Br ratio of quaternary perovskite material: (**a**–**c**) refractive index, (**d**–**f**) extinction coefficient.

**Figure 15:**
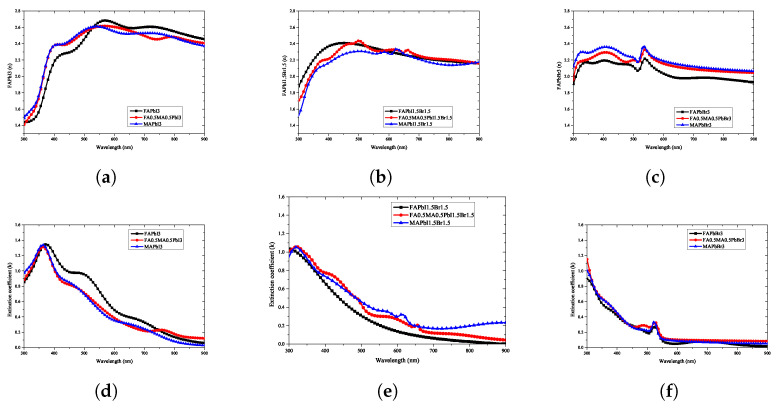
Results of optical properties with MA ratio of quaternary perovskite material: (**a**–**c**) refractive index, (**d**–**f**) extinction coefficient.

As shown in Figure 6, the optical properties corresponding to the stoichiometric ratio of the perovskite material were evaluated to have an irregular pattern. However, to find a regularity in these patterns, the change in the optical properties with the addition of Br${}_{3}$ and MA is shown in Figure 14 and Figure 15. As can be seen in Figure 14a, in FAPbI${}_{3}$ with Br${}_{3}$, the position of the characteristic peak in the 550 nm wavelength band shifted to approximately 350 nm, and the value of the refractive index decreased. This trend was confirmed in Figure 15a–c, where the change due to the addition of MA shows a similar tendency as in the case of FAPbI${}_{3}$. As can be seen in Figure 14d, the change in optical properties due to the addition of Br${}_{3}$ shows that the position of the maximum absorption peak near 380 nm was shifted to a shorter wavelength and the absorption size decreased.

In conclusion, the variation in the optical properties depending on the addition ratio of Br${}_{3}$ can be extracted through the interpolation value between the three perovskite compositions shown in Figure 14a. Although such interpolation values can be inferred, mathematical methods for extracting the accurate patterns are very limited and require complex operations. The construction of a predictive model using complex calculations for researchers wishing to study the optimal composition to improve the perovskite properties is not efficient. Therefore, it is important to develop an artificial intelligence model that can be configured with a small number of specimens by learning the measurement results using machine learning.

### 4.2. AI Training Results

To construct a hyperregression model, we predicted the optical properties of unmodified perovskite materials in the 300–900 nm range using learned artificial intelligence. Deep learning was performed using the ANN structure shown in Figure 2, and an artificial intelligence model with an optimized weighting bias was constructed. The ratios of MA, FA, I${}_{3}$, Br${}_{3}$, and wavelengths within the range of 300–900 nm were continuously input into the constructed AI model, and the predicted refractive index and extinction coefficient for each wavelength were compared with the measurement results. As compared in Figure 8, the results of the refractive index were the same for the eight stoichiometric composition ranges, but it can be seen that there was a slight difference in the case of MA${}_{0.5}$FA${}_{0.5}$PbBr${}_{3}$. As verified from Figure 8, in the case of the extinction coefficient, the results predicted through the learned artificial intelligence model were identical to the measured results. As can be seen in Figure 3b,e, the loss values of the training data set are similar to these results. However, the loss of the validation data set is 6.7654 × 10${}^{-4}$ and 2.2626 × 10${}^{-4}$ for the refractive index and extinction coefficient, respectively, and the prediction accuracy is improved by 66.5% in the extinction coefficient prediction AI. It is determined that some errors in the refractive index occurred due to the difference in the loss values of these verification data sets. It is judged that this error increases as the number of irregular patterns of the optical characteristic spectrum increases, and in the case of refractive index, since the irregularity of this spectrum is larger than the extinction coefficient, it is thought that it can be improved by collecting more specimen information. It is judged that the learning result will become increasingly accurate as the number of manufactured specimens increases. In addition, because the results discussed thus far show a very high concordance rate, we predicted the optical properties of the unmodified perovskite composition by using the trained artificial intelligence model. As shown in Figure 8, a learnt refractive index prediction AI that can calculate results similar to the experimentally analyzed optical properties was constructed. If the experimentally analyzed refractive index composition and wavelength range are input to the AI learned in this study, the results shown in Figure 9d can be obtained. These results generally have the same form as pattern recognition performed by the AI. However, in the AI model built using the hyperregression method, the interpolation value between the actually measured optical patterns can be calculated. As can be seen in Figure 9e, the refractive indices were predicted from the compositions of FA${}_{0.8}$MA${}_{0.2}$PbBr${}_{3}$ and FA${}_{0.8}$MA${}_{0.2}$PbBr${}_{3}$, which were not produced in this study. If the input composition is densely configured in the same way, the refractive index can be predicted in the FA${}_{0.9}$MA${}_{0.2}$PbBr${}_{3}$–FA${}_{0.1}$MA${}_{0.9}$PbBr${}_{3}$ range, as shown in Figure 9e. The refractive index information extracted in this way can predict changes in optical properties that depend on changes in composition, and can help determine the direction of the search for properties of an appropriate composition. As shown in Figure 9l, even in the case of the extinction coefficient, prediction is possible through the learned AI. Since the extinction coefficient has relatively few optical pattern irregularities, it can be seen that the result change of material FAMAPbI${}_{1.5}$Br${}_{1.5}$ predicted using the AI prediction model is not large. In addition, the AI model shows that the position of the characteristic peak generated near 300 nm of the MAPbI${}_{1.5}$Br${}_{1.5}$ specimen moves in the short wavelength direction as the MA composition decreases. It can be seen that the pattern of the increasing extinction coefficient seen at 700 nm or more of the MAPbI${}_{1.5}$Br${}_{1.5}$ specimen gradually decreases as the MA ratio increases. As shown in Figure 10, when examining the result of the addition of Br${}_{3}$, wherein a noticeable change in the optical characteristic is observed, it can be seen that the interpolation value of the intermediate composition is well expressed by following the irregular curve shape that occurs as the characteristic peak position changes. It can be confirmed from Figure 10a that the position of the characteristic peak of FAPbI${}_{3}$, occurring near 560 nm, shifts to approximately 350 nm with the addition of Br${}_{3}$. The magnitude of the refractive index for the maximum peak also shows a continuous decrease; hence, it can be seen that the interpolation value of the intermediate composition is well predicted for the experimental value shown in Figure 14a. As depicted in Figure 11a, the characteristic peak position occurring near 370 nm of FAPbI${}_{3}$ is shifted in the direction of 300 nm with the addition of Br${}_{3}$, and the change in the extinction coefficient, which decreases with increase in the wavelength, is predicted to decrease while maintaining the slope.

From the above results, it was possible to predict the optical properties of perovskite according to the composition through hyperregression analysis by using a trained artificial intelligence model that computes a natural interpolation value from a human cognition perspective. The variation in the optical properties of perovskite according to the stoichiometric ratio, generated through artificial intelligence, shows a very complex change in pattern, which is difficult to interpret with a general regression method. This change is well maintained even during rapid changes in the pattern according to the chemical composition. After establishing the individual hyperregression analysis model, it is possible to determine the preferred chemical composition range of the perovskite material to optimize the optical properties of the perovskite solar cell. The proposed approach has the advantage that it does not necessitate experimenting with various compositions and identifies the optimal condition within a short time by preferentially experimenting in the target composition range. In addition, the amount of big data used for the hyperregression analysis can be increased by conducting experiments with the predicted composition, and a more sophisticated predictive model can be created by performing additional learning using the newly collected data.

## 5. Conclusions

In this study, the optical properties of perovskite materials according to the stoichiometric ratio were measured using ellipsometry, and the measured results were analyzed using an optical simulation model. The refractive index and extinction coefficient extracted through the ellipsometry analysis showed a tendency consistent with the color change of the specimen, and had shapes similar to those reported in the literature. The machine learning method was used for hyperregression analysis of the data with five variables: four compositional variables of the perovskite material and one wavelength variable. An ANN structure was constructed to enable hyperregression analysis of n-dimensional variables, and deep learning was performed by updating the weights and bias values of the neural network structure by using gradient descent and error backpropagation methods. Finally, by inputting the actually fabricated stoichiometric ratio and wavelength range to the learned artificial intelligence model, it was confirmed that the optical properties were similar to those measured with an ellipsometer. However, the loss of the validation data set is 6.7654e-4 and 2.2626e-4 for the refractive index and extinction coefficient, respectively, and the prediction accuracy is improved by 66.5% in the extinction coefficient prediction AI. It is judged that this error increases as the number of irregular patterns of the optical characteristic spectrum increases, and in the case of the refractive index, since the irregularity of this spectrum is larger than the extinction coefficient, it is thought that it can be improved by collecting more specimen information. When the optical properties of unmodified perovskite were predicted using the verified artificial intelligence model, a very complex pattern change was observed, which was impossible to analyze using a general regression method. This change is well maintained, even in a pattern that rapidly changes according to the change in composition. In conclusion, hyperregression analysis with n-dimensional variables can be performed using a simple big data construction method for the spectral patterns of thin-film materials. This analytical technique is considered to greatly reduce the parameters in future experiments because it is possible to perform hyperregression analysis and spectral analysis with a single parameter, such as energy band gap or electron affinity.

## Figures and Tables

**Table 1:** Molar concentrations of the precursors in the perovskite precursor solution of each composition.

Perovskite Compositions	Molar Concentration of Precursors in Respective Compositions					
	MAI	MABr	FAI	FABr	PbI${}_{2}$	PbBr${}_{2}$
FAPbI${}_{3}$	0	0	1.14	0	1.25	0
FAPbI${}_{1.5}$PbBr${}_{1.5}$	0	0	0.57	0.57	0.625	0.625
FAPbBr${}_{3}$	0	0	0	1.14	0	1.25
FA${}_{0.5}$MA${}_{0.5}$PbI${}_{3}$	0.57	0	0.57	0	1.25	0
FA${}_{0.5}$MA${}_{0.5}$PbI${}_{1.5}$PbBr${}_{1.5}$	0.285	0.285	0.285	0.285	0.625	0.625
FA${}_{0.5}$MA${}_{0.5}$PbBr${}_{3}$	0	0.57	0	0.57	0	1.25
MAPbI${}_{3}$	1.14	0	0	0	1.25	0
MAPbI${}_{1.5}$PbBr${}_{1.5}$	0.57	0.57	0	0	0.625	0.625
MAPbBr${}_{3}$	0	1.14	0	0	0	1.25

**Table 2:** Big data of refractive index and extinction coefficient composition details according to the optical properties of perovskite materials by stoichiometric ratio.

Count	Big data of refractive index					
	Input neuron data					Output neuron data
	MA	FA	I${}_{3}$	Br${}_{3}$	Wavelength (μm)	n
1	1	0	1	0	0.976372547	2.406349547
2	1	0	1	0	0.975582775	2.406770478
⋮	⋮	⋮	⋮	⋮	⋮	⋮
928	1	0	1	0	0.244254811	1.523567095
929	1	0	0.5	0.5	0.976372547	2.141620475
⋮	⋮	⋮	⋮	⋮	⋮	⋮
6612	0	1	0	1	0.244254811	1.501819505
Count	Big data of extinction coefficient					
	Input neuron data					Output neuron data
	MA	FA	I${}_{3}$	Br${}_{3}$	Wavelength (μm)	k
1	1	0	1	0	0.976372547	0.029159807
2	1	0	1	0	0.975582775	0.029408273
⋮	⋮	⋮	⋮	⋮	⋮	⋮
928	1	0	1	0	0.244254811	0.741038184
929	1	0	0.5	0.5	0.976372547	0.021949375
⋮	⋮	⋮	⋮	⋮	⋮	⋮
6612	0	1	0	1	0.244254811	0.754939147

## Data Availability

Not applicable.

## Abbreviations

The following abbreviations are used in this manuscript:

ANN	Artificial Neural Network
MA	Methylammonium (CH${}_{3}$NH${}_{3}$)
FA	Formamidinium (CH(NH${}_{2}$)${}^{2+}$)
FDTD	Finite Difference Time Domain
CNN	Convolutional Neural Network
ITO	Indium-doped Tin Oxide
DMF	Dimethyl Formamide
r DMSO	Dimethyl Sulfoxide
SEM	Scanning Electron Microscopy
CVD	Chemical Vapor Deposition
EMA	Effective Medium Approximation
ReLU	Rectified Linear Unit
MSE	Mean Squared Error
